# Numerical Investigation of Tunable Plasmonic Tweezers based on Graphene Stripes

**DOI:** 10.1038/s41598-017-14912-w

**Published:** 2017-11-06

**Authors:** Mohsen Samadi, Sara Darbari, Mohammad Kazem Moravvej-Farshi

**Affiliations:** Faculty of Electrical and Computer Engineering, Tarbiat Modares University, P. O. Box 14115-194, Tehran, 1411713116 Iran

## Abstract

We are proposing tunable plasmonic tweezers, consisting two parallel graphene stripes, which can be utilized to effectively trap and sort nanoparticles. We show that by electrostatically tuning the chemical potential of a graphene stripe by about 100 meV (equivalent to Δ*V*_G_ ≈ 4.4 V), the plasmonic force can be switched efficiently, without a need to switch the laser intensity. This enables high speed and low power switching with a large number of switching cycles. By applying two independent and appropriate gate bias voltages to the stripes, the direction of the plasmonic force can be reversed, which leads to separation of nanoparticles that satisfy the trapping conditions. Numerical simulations show that the potential depths obtained for polystyrene nanoparticles of refractive index *n* = 1.5717 and radii *r* ≥ 50 nm is deeper than −10 *k*_B_ *T* , confirming the ability of the proposed system to effectively separate such nanoparticles. This capability holds for smaller nanoparticles with larger refractive indices. Finally, performing thermal simulations, we have demonstrated that the heat induced by the illumination increases the fluid temperature by at most 9 °C, having negligible effect on the trapping mechanism. The proposed system opens up new possibilities in developing tunable on-chip manipulation devices, suitable for biological applications.

## Introduction

Optical manipulation has been shown to be one of the most suitable approaches for trapping and sorting of micron sized particles and biological cells, due to its high efficiency, low cost and non-invasive nature of light in manipulating particles^1^. Trapping of nanometer sized particles has been reported before^2,3^. However, as particle size decreases, the magnitude of the trapping force drops with the third power of its radius^4^, making stable trapping of sub-micrometer particles difficult. One might think of increasing the incident optical power as a way to upturn the optical force. However, the temperature increase due to the power absorption in an aqueous environment may damage the particles. An alternative approach to amplify the optical force is to focus the same incident power into a very small spot, causing an extremely high power density. However, optical diffractions limit the spot size into which the incident beam can be focused. Hence, the conventional optical tweezers are only applicable for manipulation of particles of dimensions greater than the diffraction limits^3^.

Recently, the near-field optical manipulation methods have been shown to be a promising solution to overcome the diffraction limits^5–7^. Among the near-field methods, plasmonic particle manipulation has received a lot of attentions, owing to sub-diffraction limit field confinement and strong field enhancement at metal/dielectric interface^4,8–19^. Numerous studies on plasmonic manipulation have utilized Kretschmann configuration to excite surface plasmons (SPs), in which the gold/water interface is illuminated through a glass prism, at a specific angle and polarization^15,18,19^. One major drawback of using metallic structures is the significant loss in metals and the related extreme heat generation, which may damage the biological cells at high incident powers in biological applications.

Graphene, a 2D lattice of carbon atoms, has been proposed as a good alternative plasmonic material^20–22^, owing to low optical loss and tunable optical properties^23–25^. Hence, the field enhancement factor can be controlled by electrostatic gating of graphene at an appropriate fixed incident laser intensity^26,27^. Considering this tunable plasmonic behavior, we have recently proposed graphene as an attractive candidate for plasmonic tweezers, for the first time^28^. Furthermore, graphene’s high thermal conductivity as compared with metals^25^ causes the heat generated by the absorbed incident power to be removed efficiently. This property can be vital for manipulating biological samples.

Here, we have designed a plasmonic force switch in mid-IR range, benefiting from surface plasmons in graphene stripes for sorting nanoparticles.

### The Proposed Structure and Operation Principle

**Figure 1:**
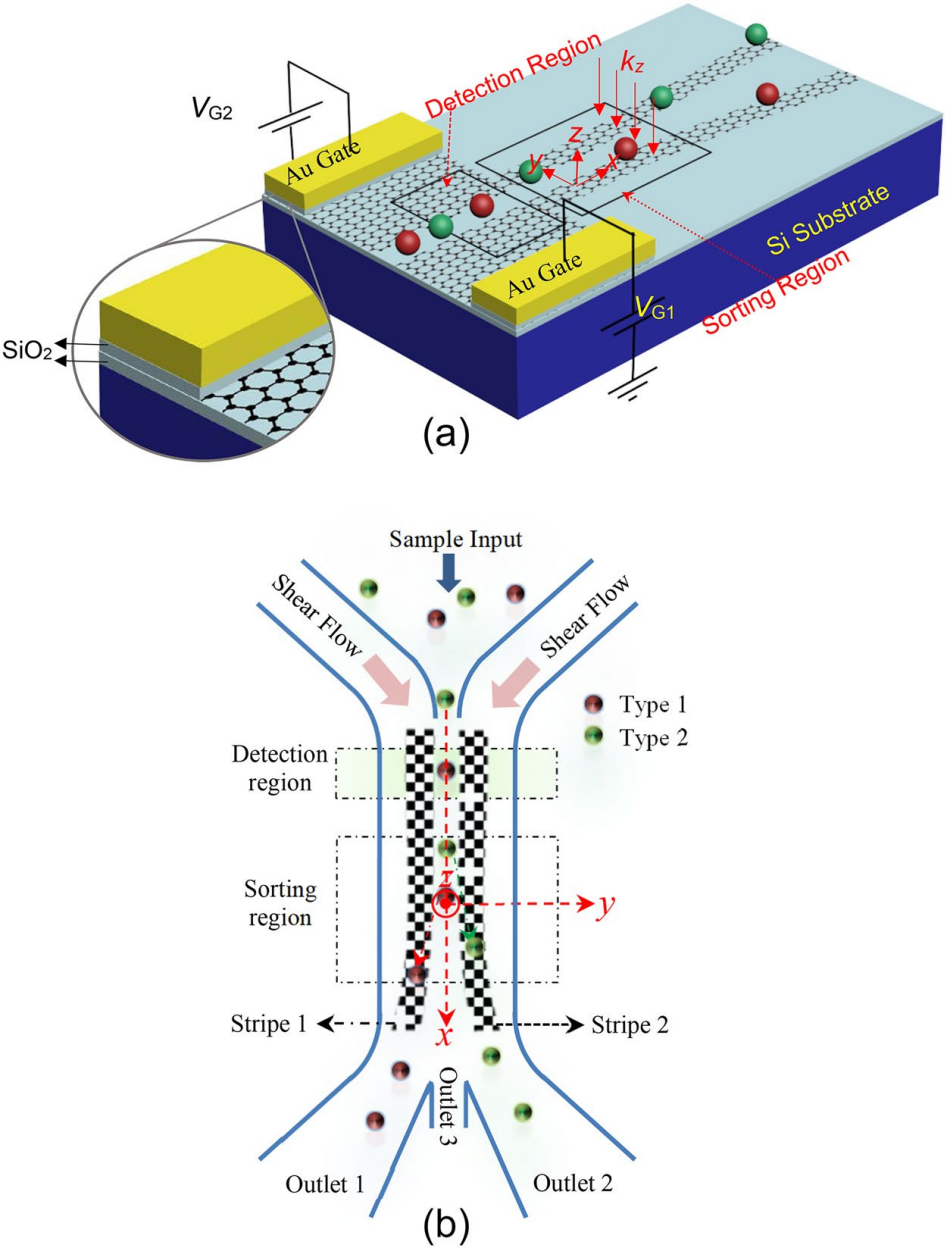
Schematic of the proposed system: (**a**) 3D and (**b**) Top views.

Figure 1 shows a 3D schematic representation (a), and top view (b) of the proposed system. It consists of two 100 nm wide graphene stripes on SiO_2_, separated by a 100 nm spacing. This spacing isolates the stripes electrostatically and enables us to control their chemical potential independently by applying different gate bias voltages (${V}_{{\text{G}}_{1,2}}$). The zoomed-in view displays the structure cross-section, in which each of the two graphene stripes is topped by a 10–nm thick SiO_2_ layer that separates the stripe from its top gold (Au) gate contact. As can be seen from the figure, the system is divided into two regions - i.e., the detection and the sorting regions. Polystyrene particles are tagged by two different fluorescence labels (red and green), before being injected into the microfluidic channel via the sample inlet. The injected particles are focused, hydrodynamically, to a line of single particles moving along the channel midline^5,19,29^. Then, they are envisioned by fluorescence microscopy while passing through the detection region^28^, where they can be identified by their colored labels. Next, in the sorting region, appropriate bias voltages (${V}_{{\text{G}}_{1}}$ and ${V}_{{\text{G}}_{2}}$) are applied to the graphene stripes to adjust the chemical potentials, according to the data achieved from the detection region. Meanwhile, graphene stripes are normally illuminated in the sorting region, by a plane wave with linear polarization along the stripes widths. For the red (green) particles, ${V}_{{\text{G}}_{1}}$ (${V}_{{\text{G}}_{2}}$) is switched on, so that the field enhancement occurs on the stripe 1 (2), where the localized SPs are excited. We intentionally chose the polarization direction of the incident light to be along the stripes widths, so that the localized SPs are excited due to the confinement of the graphene stripes in y-direction. Since the incident light is illuminated normally and the *x* component of the wave vector is zero (*k*_*x*_ = 0), the generated localized SPs are not expected to propagate along the *x* axis. Hence, the optical force has no *x*-component to affect the particles movement along the *x*-direction. The gradient component of the plasmonic force pushes down the red (green) particles toward the stripe 1 (2) while passing through the sorting region, until they exit from the outlet 1 (2). Nonetheless, if the trapping conditions are not satisfied, the untrapped particles continue to move along the channel midline (*x*-direction) without deflection, exiting from outlet 3. The proposed design enables us to electrostatically switch the direction of gradient force and sort the particles according to their fluorescent labels. This technique allows the force switching by a low voltage electrical gating, without a need to switch the laser intensity. High speed and low power switching, in addition to enabling a large number of switching cycles, are among other advantages of this configuration.

### Simulation Method

To elaborate the proposed system quantitatively, numerical simulations were conducted using 3D finite difference time domain technique, using the perfectly matched layer boundary condition. Graphene is considered to be a single 2D layer, whose optical surface conductivity can be derived using Dyadic Green’s function^30^:

1 $$\sigma (\omega ,{\mu }_{C},{\rm{\Gamma }},T)=\tfrac{j{e}^{2}(\omega -j2{\rm{\Gamma }})}{\pi {\hslash }^{2}}[\tfrac{1}{{(\omega -j2{\rm{\Gamma }})}^{2}}\,{\int }_{0}^{\infty }\,E(\tfrac{\partial {f}_{d}(E)}{\partial E}-\tfrac{\partial {f}_{d}(-E)}{\partial E})\,dE-{\int }_{0}^{\infty }\,\tfrac{{f}_{d}(-E)-{f}_{d}(E)}{{(\omega -j2{\rm{\Gamma }})}^{2}-4{(E/\hslash )}^{2}}dE],$$

where *e* is electron charge, $\hslash$ is the reduced Planck’s constant, ${f}_{d}(E)={[{e}^{(E-{\mu }_{c})/{k}_{B}T}+1]}^{-1}$ is the Fermi-Dirac distribution, *k*_B_ is the Boltzmann’s constant, *ω* is the angular frequency, *μ*_*c*_ is the graphene chemical potential, ${\rm{\Gamma }}=e{{v}_{f}}^{2}/2{\mu }_{e}{\mu }_{c}$ is the phenomenological scattering rate, *μ*_*e*_ is the electron mobility, ${{\upsilon }}_{f}\approx {10}^{6}\,\text{m/s}$ is the Fermi velocity in graphene, and *T* is the temperature. The first and the second integrals in Equation 1 refer to the intraband and interband transitions, respectively. It can be observed that optical surface conductivity of graphene, therewith its plasmonic behavior, can be controlled by modulating *μ*_*c*_, via electrostatic gating.

The gate voltage ${V}_{{\text{G}}_{1}}$ (${V}_{{\text{G}}_{2}}$) needed to create appropriate chemical potential in a graphene stripe is calculated using a simple parallel plate capacitor model^31^. According to this model, the applied voltage *V* changes the charge carrier density *n* of a graphene stripe:

2 $$\begin{array}{l}n=\frac{{\varepsilon }_{0}{\varepsilon }_{d}}{{t}_{d}}(V+{V}_{0})\end{array}$$

where *ε*_*d*_ and *t*_*d*_ are the dielectric constant and the thickness of the gate oxide and *V*_0_ is the offset voltage caused by natural doping. This leads to a shift in the chemical potential of the graphene stripe:

3 $$\begin{array}{l}{\mu }_{c}=\hslash {v}_{f}\sqrt{\pi n}\end{array}$$

From Equations 2 and 3, the gate voltage needed to create a determined chemical potential in each of the stripes is calculated:

4 $$\begin{array}{l}V=\frac{{t}_{d}}{{\varepsilon }_{0}{\varepsilon }_{d}}.\frac{{\mu }_{c}^{2}}{\pi {\hslash }^{2}{v}_{f}^{2}}\end{array}$$

The average optical force exerted on a particle is evaluated using the surface integral:

5 $$\begin{array}{l}\langle F\rangle =\frac{1}{2}{\rm{Re}}\,{\oint }_{{\rm{\Omega }}}\,{\bf{T}}({\bf{r}},t)\cdot \widehat{{\bf{n}}}\,ds\end{array}$$

6 $$\begin{array}{l}{\bf{T}}({\bf{r}},t)=\varepsilon {\bf{E}}({\bf{r}})\otimes {{\bf{E}}}^{{\boldsymbol{\ast }}}({\bf{r}})+\mu {\bf{H}}({\bf{r}})\otimes {{\bf{H}}}^{{\boldsymbol{\ast }}}({\bf{r}})-\frac{1}{2}(\varepsilon {|{\bf{E}}({\bf{r}})|}^{2}+\mu {|{\bf{H}}({\bf{r}})|}^{2})\end{array}$$

where ${\bf{T}}({\bf{r}},t)$ is the Maxwell stress tensor, *ε* and *μ* are the medium permittivity and permeability, **E** and **H** are the electric and magnetic field intensity vectors, **r** and *t* represent the position vector and time, and **n** is the unitary vector normal to the surface that encloses the volume Ω. This volume is assumed to be a cubic box surrounding the particle that moves along with the particle in order to consider the displacements.

## Numerical Results and Discussions

**Figure 2:**
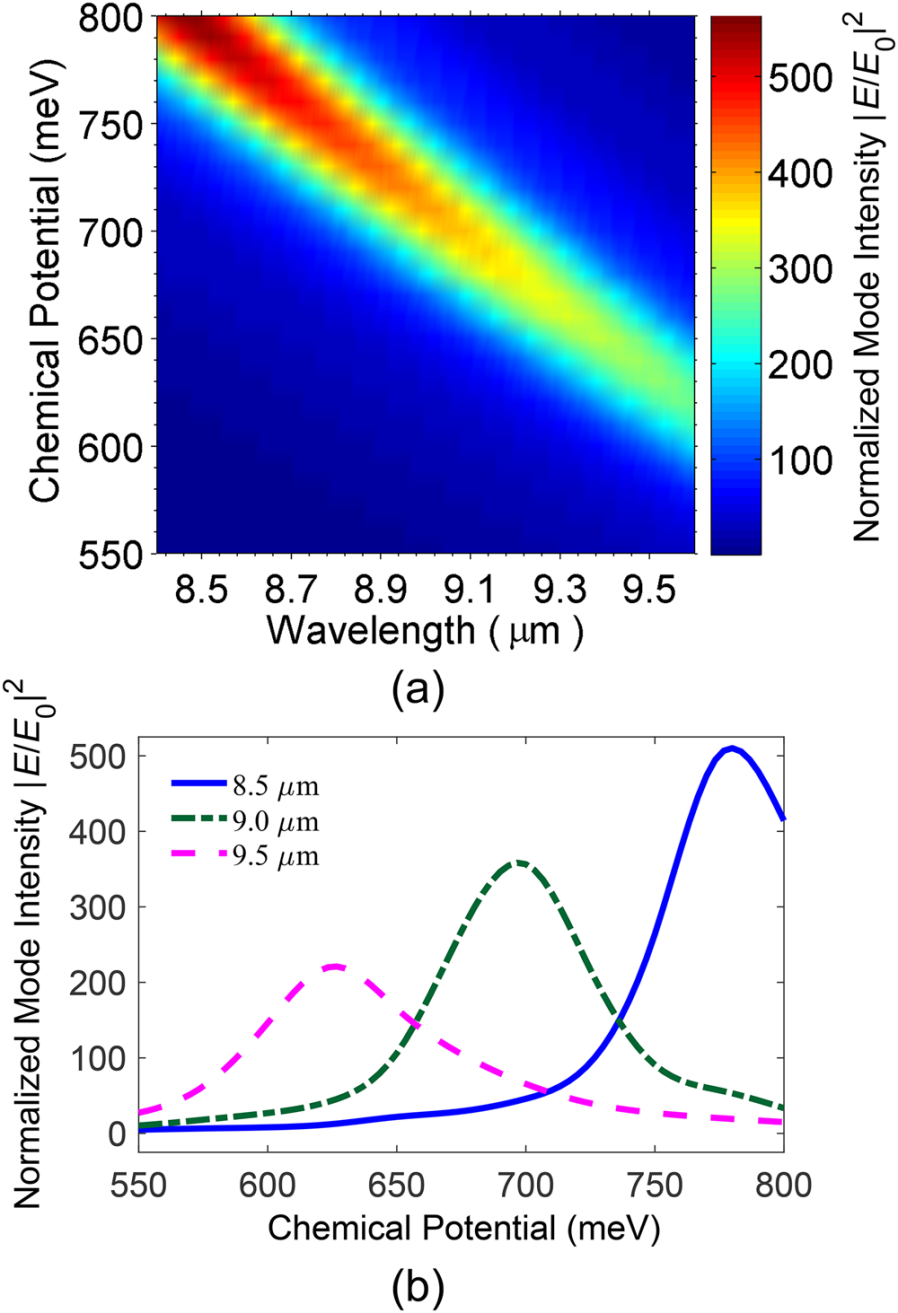
Normalized mode intensity map, versus chemical potential and incident wavelength. (**b**) Normalized mode intensity versus chemical potential, for *λ* = 8.5, 9.0, and 9.5 μm.

To design an efficient plasmonic force system, first, we studied the effect of different system’s parameters on the plasmonic field distribution. For this purpose, as we have pointed out earlier, a laser light is illuminated to a single graphene stripe at normal incidence. The laser light is assumed to be a plane wave with linear polarization along the width of the stripe. The substrate and the fluid containing polystyrene nanoparticles are considered to be glass (*n*_*g*_ = 1.52) and water (*n*_*w*_ = 1.33), respectively. Figure 2(a) shows mode intensity map normalized with respect to the incident light intensity (${|E/{E}_{0}|}^{2}$), probed at 10 nm above the graphene stripe, as the incident wavelength and the chemical potential are varied (8.4 ≤ *λ* ≤ 9.6 μm and 550 ≤ *μ*_*c*_ ≤ 800 meV). As can be observed in this figure, the electromagnetic field is enhanced due to surface plasmon excitation at specific wavelengths and chemical potentials.

Figure 2(b) displays the normalized mode intensity profiles for three different wavelengths versus the chemical potential. As shown in this figure, by tuning the chemical potential, at any of the three given wavelengths, the related field intensity can be switched from its minimum to maximum value and vice versa. Henceforth, we assume the incident wavelength to be 9 μm, so we can simply switch between its ON state (nearly maximum field intensity) and OFF state (less than 10% of the maximum) by switching the chemical potential between 700 and 600 meV respectively. Considering the 10-nm thick SiO_2_ as the gate oxide and using Equation 4, one can easily show that a difference of ${\rm{\Delta }}{V}_{{\rm{G}}}\approx 4.4\,{\rm{V}}$ in the applied gate voltage can lead to 100 meV shift in the chemical potential of the graphene stripe.

**Figure 3:**
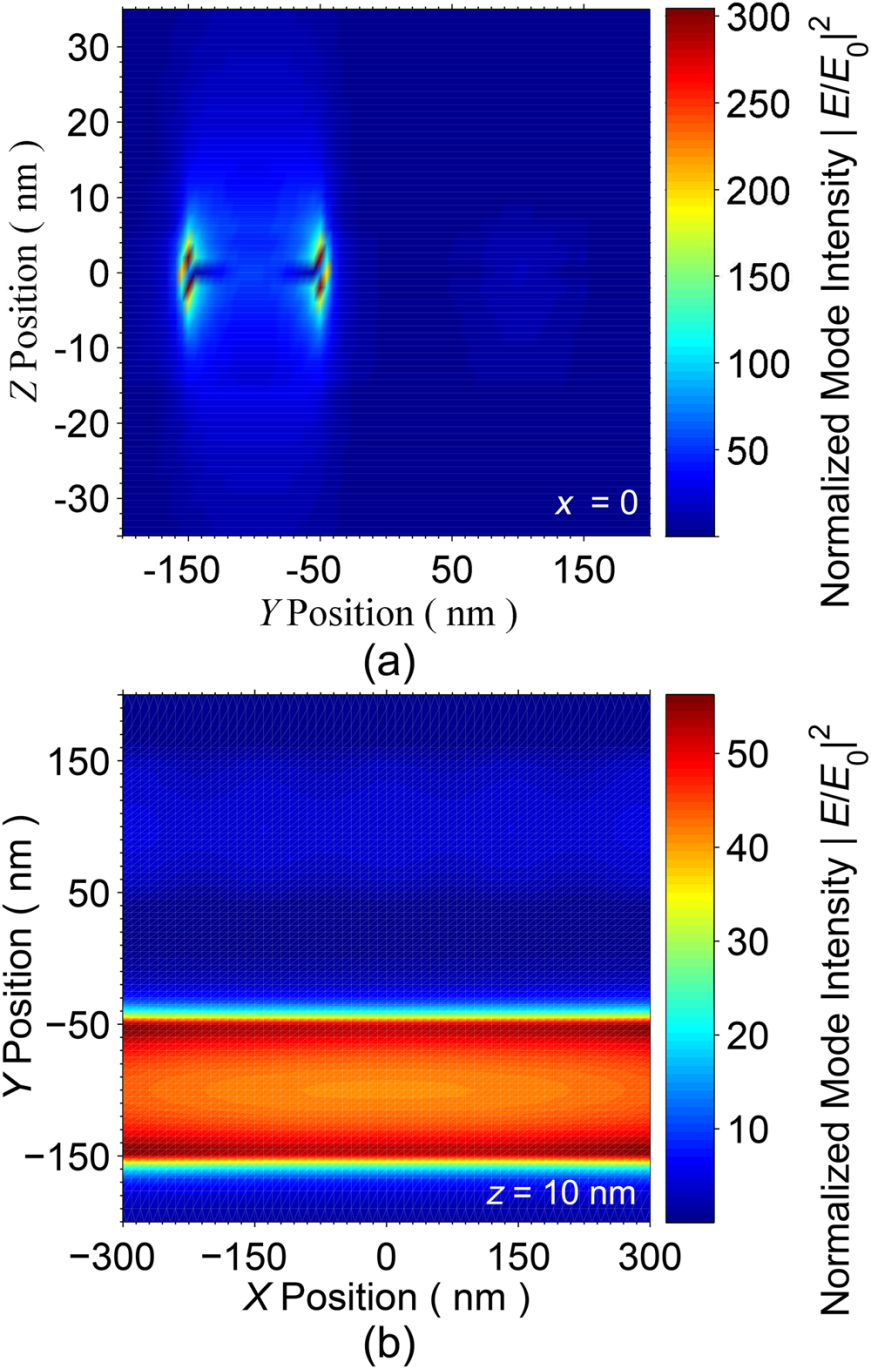
Field distribution in (**a**) *y* − *z* plane (at *x* = 0) and (**b**) *x* − *y* plane (*z* = 10 nm), when stripe 1 is ON and stripe 2 is OFF.

To investigate the sorting functionality of our proposed double graphene stripe system, the normalized field distribution in the *y* − *z* plane (at *x* = 0) and in the *x* − *y* plane (at $z=10\,\text{nm}$) are calculated. The results are shown in Fig. 3(a),(b), respectively. As can be observed from these figures, the bias voltages ${V}_{{{\rm{G}}}_{1}}$ and ${V}_{{{\rm{G}}}_{2}}$ are adjusted such that the stripe 1 is ON and the stripe 2 is OFF. In other words, the SPs are excited on the surface of the stripe 1. The results for the case, in which the metallic gates are biased such that the stripe 1 is OFF and the stripe 2 is ON are the mirror images of those shown in Fig. 3(a),(b), with respect to the $x-z$ plane (*y* = 0). In the latter case, the particles are pulled towards the stripe 2. As shown in Fig. 3, the field intensity is non-uniform across the stripe width (y-direction). It is maximum near the edges and sharply drops across the stripe width. Meanwhile, the same data reveal that the field intensity along the stripe (*x*-direction) is uniform. Thus, the plasmonic gradient force along the stripe is expected to be zero. Besides, since the wave vector of the incident beam has no *x*-component, we expect the *x*-component of the net plasmonic force to be zero.

**Figure 4:**
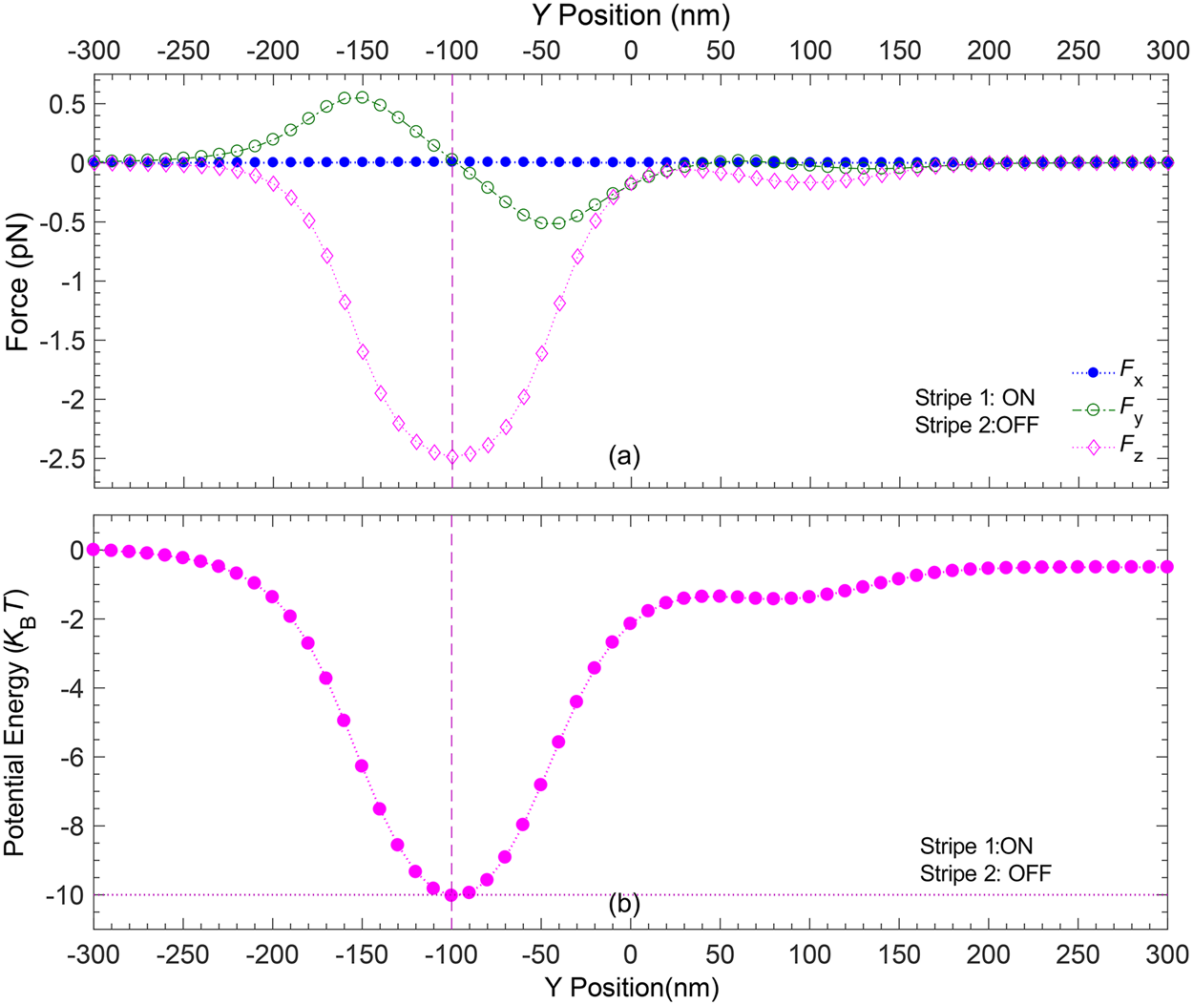
(**a**) Plasmonic force components exerted on a polystyrene nanoparticle (**b**) Trapping potential, when stripe 1 is ON and stripe 2 is OFF. The vertical dashes *y* = −100 nm show the trapping y-positions.

For an incident light of intensity $I=20\,\text{mW}/\mu {\text{m}}^{2}$, the components of the force exerted on a polystyrene nanoparticle of radius $r=50\,\text{nm}$ and refractive index $n=1.5717$ are calculated. The results for the biasing condition in which stripe 1 is ON and stripe 2 is OFF are shown in Fig. 4(a). Those for the biasing condition in which stripe 1 is OFF and stripe 2 is ON are the mirror images of the data shown in Fig. 4(a), with respect to $y=0$. As shown in this figure, the z-component of the plasmonic force above the stripe 1 is negative (${F}_{z} < 0$), pushing the nanoparticle toward the graphene surface with a peak strength (|*F*_*z*_|_max_ ≈ 2.5 pN) located at the stripe midline ($y=-100\,\text{nm}$ indicated by the vertical dashes). Moreover, this figure shows that as one moves across the width of the stripe 1, from the left to the right of the horizontal axis, the y-component of the plasmonic force (*F*_*y*_) starts from its maximum value (|*F*_*y*_|_*max*_ ≈ 0.55 pN) at the stripe left edge $(y=-150\,\text{nm})$, decreasing toward zero at the stripe midline where the nanoparticle might be trapped, and continue to decrease until it reaches its largest negative value $({F}_{{y}_{{\min }}}=-{F}_{{y}_{{\max }}})$ at the stripe right edge $(y=-50\,\text{nm})$. As a consequence, a potential well across the stripe 1 is formed that can trap the particle above the stripe, if equals $-10\,{k}_{\text{B}}T$ or deeper. Moreover, the blue bullets in Fig. 4(a) show that the x-component of the plasmonic force is zero (${F}_{x}=0$), allowing the fluid flow to push the trapped particle toward the outlet 1 for this case. When the biasing condition is reversed, the potential formed across the stripe 2 may trap the particle above the stripe, and the fluid flow pushes the trapped particle toward the outlet 2. If the potential depth is, however, shallower than $-10\,{k}_{\text{B}}T$, the trapping condition is not fulfilled and fluid flow pushes the untrapped particles toward the outlet 3.

Figure 4(b) shows the profiles of the potential energies along the y-direction for the same biasing condition as for Fig. 4(a). These profiles are obtained by the integral $\int {F}_{y}dy$ above and across the graphene stripes widths. As shown in this figure, when the intensity of the incident light is $I=20\,\text{mW}/\mu {\text{m}}^{2}$, the depth of the potential well is slightly deeper than $-10\,{k}_{\text{B}}T$, enough for a stable trap that can overcome the thermal oscillations of particles^1^. The trapped particles y-positions, for the given operating condition are indicated by the vertical dashes. As explained earlier, the only force affecting the particles movements along the channel is due to the fluid flow. Hence, the trapped particles move along the stripe that is ON, before exiting from the corresponding outlet 1(2).

**Figure 5:**
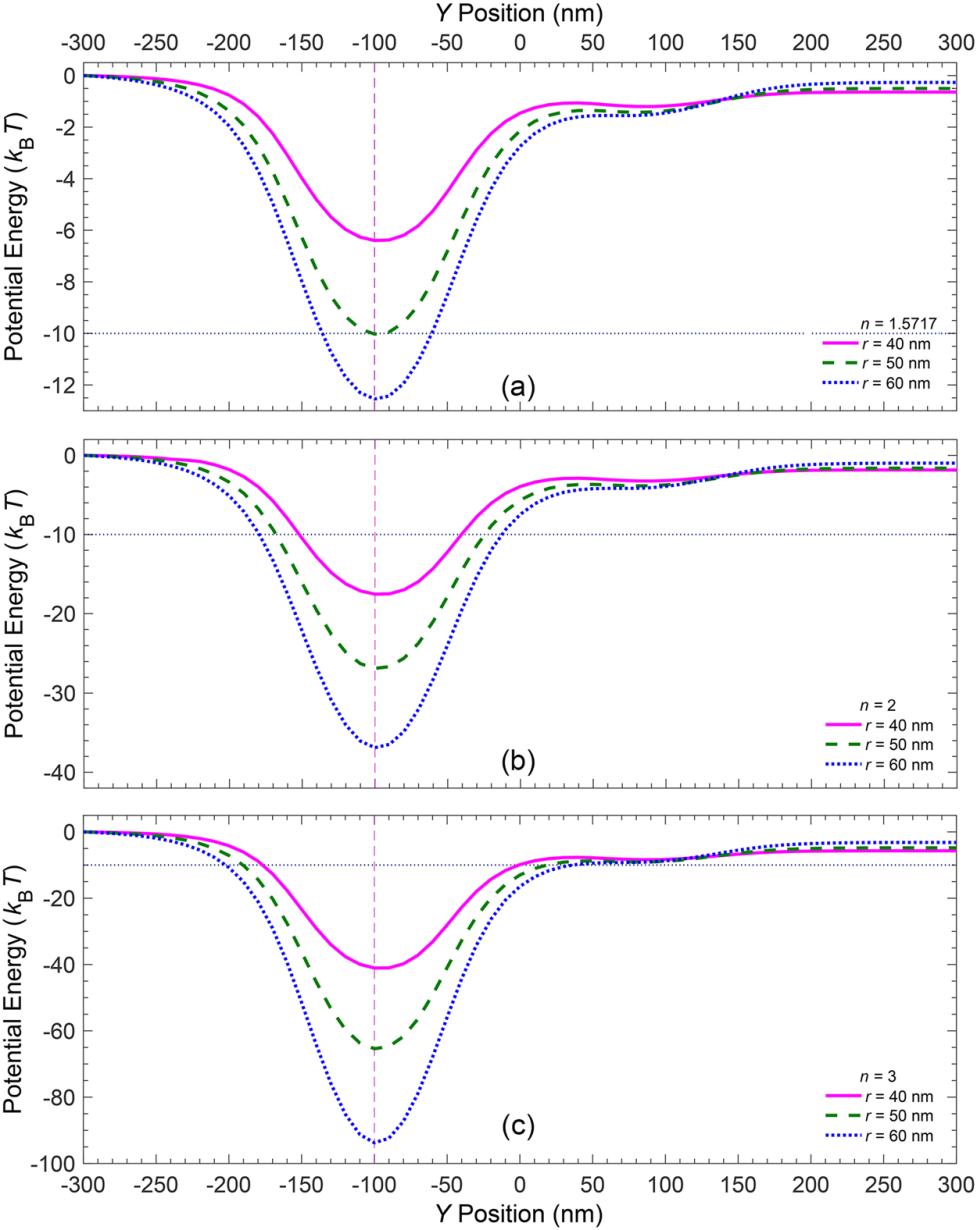
Potential energies, across the width of the sorting region, for nanoparticles of radii *r* = 40, 50, *and* 60 *nm* and the refractive index of (**a**) $n=1.5717$, (**b**) *n* = 2, and (**c**) *n* = 3.

Next, we have investigated the effect of the particle size and refractive index on trapping conditions. Figure 5 illustrates the potential energies versus the y-position across the width of the sorting region, for nanoparticles of different radii and refractive indices under the same operating conditions as for Figs 3 and 4. The numerical results for three polystyrene nanoparticles $(n=1.5717)$ of radii $r=40,\,50,\,\text{and}\,60\,\text{nm}$ are shown in Fig. 5(a). It can be observed that the larger the particle radius, the stronger the plasmonic force exerted on it and hence the deeper the resulting potential well across the stripe that is ON. However, there is a limit in increasing the particle size. As the particle’s diameter becomes larger than the stripe width, the portion of the particle volume that can effectively interact with the plasmonic field is decreased, decreasing the potential depth as a consequence. Moreover, Fig. 5(a) also reveal that the given operating condition in the presence of polystyrene nanoparticles with radii $r < 50\,\text{nm}$ cannot create potential wells deep enough to trap the particles effectively. Figure 5(b,c) illustrate the numerical results obtained for similar nanoparticles with refractive indices of $n=2\,\text{and}\,3$, respectively. Comparison of potential profiles for particles of the same radii but different refractive indices demonstrates that the larger the refractive index, the stronger the plasmonic force exerted on the particle and the deeper the resulting potential well across the stripe that is ON.

**Figure 6:**
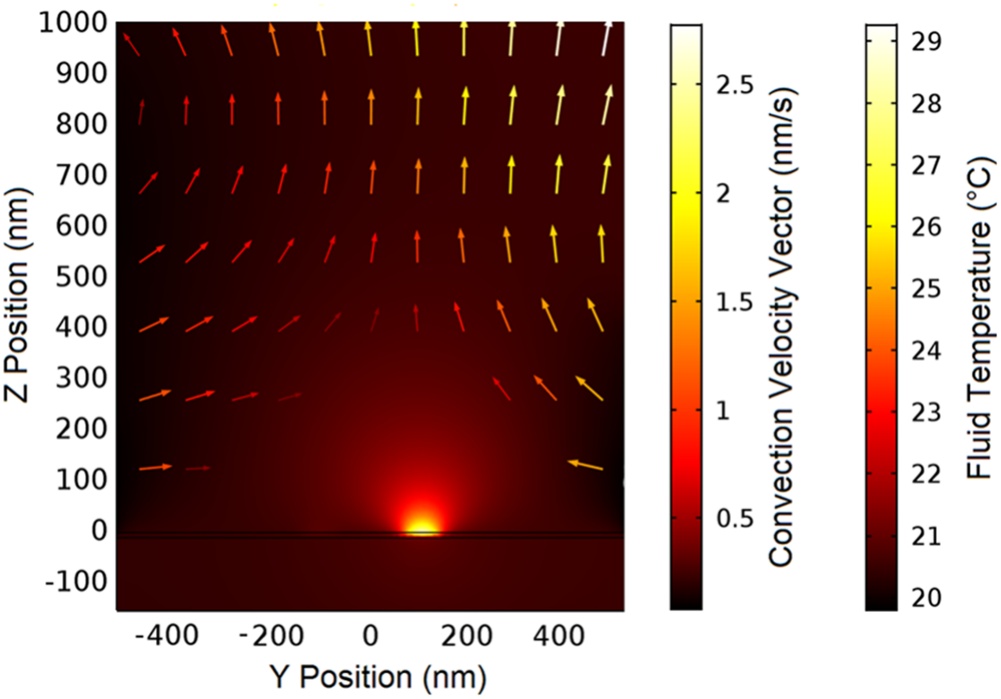
Spatial distribution of the temperature and fluidic convection velocity vectors (arrows) in an aqueous medium above the graphene stripes, when the stripe 1 in ON and *I* = 20 mW/μm^2^.

The laser intensity used for the stable trapping in this research is comparable to that of the recently proposed graphene-based plasmonic tweezers^22^ as well as other metal plasmonic structures^32–34^. Higher intensities have also been used with the aid of appropriate heat sinks to overcome the thermal effects^35^. Moreover, high thermal conductivity $(2000\,{\text{Wm}}^{-1}{\text{K}}^{-1}$ of graphene as compared to that of gold ($320\,{\text{Wm}}^{-1}{\text{K}}^{-1}$) in addition to its extremely low power absorption quickly removes the generated heat^36^. It has also been numerically shown that the same laser intensity generates much less heat in graphene surrounding nanoholes than it generates in a gold layer surrounding nanoholes^21^. Nonetheless, to elaborate the thermal behavior of the proposed system, we have carried out the heat transfer and convection calculations in the $y-z$ plane $(x=\mathrm{0)}$, using finite element method with open boundary condition at ${T}_{0}=20\,{}^{^\circ }\text{C}$. We also considered no-slip boundary condition at *z* = 0 that assumes a zero fluid velocity close to the interface between the fluid and the substrate. Colored pattern in Fig. 6 represents the spatial distribution of the fluid temperature and the arrows directions and colors together define the fluidic convection velocity vectors, when the stripe 1 in ON and $I=20\,\text{mW}/\mu {\text{m}}^{2}$. As can be observed from this figure, the maximum temperature above the stripe 1 that is ON reaches to 29 °*C*.

Using the data shown in Fig. 6, we can estimate the stochastic Langevin force, ${F}_{th}$ that is responsible for Brownian motion of the particles, from its correlation function, $\langle {F}_{th}(t){F}_{th}(t^{\prime} )\rangle =2{k}_{B}T\gamma \delta (t-t^{\prime} ).$ Here, ${k}_{B}$, *T*, and $\gamma =6\pi \eta r$ are the Boltzmann constant, the fluid temperature, and the drag coefficient, in which $\eta$ is the fluid viscosity. The delta function $\delta (t-t^{\prime} )$ states that there is no correlation between the force in time t and any other instants of time^18,37^. As an example, the maximum Langevin force that may be exerted on a 50-nm particle with the achieved thermal conditions (Fig. 6) is on the order of a few fNs that is about three orders of magnitude smaller than the calculated optical force maxima. Moreover, the fluidic convection velocity vectors, shown in Fig. 6, indicate that the temperature gradient leads to a convective fluid flow around the bright spot that may adversely affect the trapping and sorting operation. However, the effective force on a spherical particle due to this convective flow has been obtained using the Stokes’ drag force equation ${F}_{D}=\gamma v$, in which $v$ is the fluidic convection velocity vector^38^. Considering the maximum velocity observed in Fig. 6, the effective force due to the convection is also negligible as compared with the optical force maxima. Also, in a spatial temperature gradient, a particle is subjected to thermophoretic force that can be approximately determined by ${F}_{T}=-\gamma {v}_{T}$. Here, ${v}_{T}=-{D}_{T}\nabla T$ is the steady state thermophoretic velocity acquired by a particle drifting along the temperature gradient $\nabla T$, and *D*_*T*_ is the thermophoretic mobility^18,38,39^. The amounts of *D*_*T*_ for different aqueous particle suspensions have been measured in numerous experiments and is reported to vary in a range within $1 < {D}_{T} < 10\,\mu {\text{m}}^{2}\,{{\rm{s}}}^{-1}\,{{\rm{K}}}^{-1}$ ^40^. Specifically, for polystyrene spheres of radii $r\approx 50\,{\rm{n}}{\rm{m}}$ immersed in water, ${D}_{T}\approx 1.55\,{\mu {\rm{m}}}^{2}\,{{\rm{s}}}^{-1}\,{{\rm{K}}}^{-1}$. Assuming a uniform temperature gradient of ${\rm{\nabla }}T=10\,{\rm{K}}{\mu {\rm{m}}}^{-1}$, the thermophoretic force becomes about two orders of magnitude smaller than the optical force maxima^41^. Hence, these infinitesimally negligible Langevin, thermophoretic and Stokes’ drag forces has insignificant effects on the performance of the designed plasmonic tweezers.

## Conclusion

In this paper, we have presented a tunable plasmonic nanoparticle sorting system based on two parallel graphene stripes. Each of the 100-nm wide graphene stripes has been biased independently, to control its chemical potential via electrostatic gating. Simulations show that by altering the chemical potentials of the two stripes, the direction of the plasmonic force exerted on nanoparticles moving in the microfluidic channel is reversed. Injected polystyrene particles, which are hydrodynamically focused in the centerline of the channel, are separated by appropriate electrical gating and are collected through either of the two designated outlets, if the trapping conditions are satisfied. Otherwise, the untrapped particles continue to move with the fluid along the channel midline, flowing out from the third outlet. It is shown that altering the gate voltage about 4.4 V results in potential depth variation of about −10 *k*_B_ *T* for a polystyrene nanoparticle of radius $r=50\,{\rm{n}}{\rm{m}}$ and refractive index $n=1.5717$. The potential depth and hence, the trapping stability can be improved by using larger polystyrene nanoparticles or smaller nanoparticles with higher refractive indices. Moreover, performing thermal simulations have shown that the heat induced by the 20 mW/μm^2^ illumination elevates the fluid temperature by at most 9 °C that has infinitesimally negligible effect on the plasmonic trapping mechanism. The proposed design enables us to electrically switch the direction of the exerted plasmonic force and actively sort the particles.
